# The development of the International Classification of Functioning, Disability and Health Core Sets for deafblindness: A study protocol

**DOI:** 10.1371/journal.pone.0261413

**Published:** 2021-12-14

**Authors:** Abinethaa Paramasivam, Atul Jaiswal, Renu Minhas, Peter Holzhey, Karen Keyes, Ricard Lopez, Walter Wittich

**Affiliations:** 1 School of Optometry, Université de Montréal, Montreal, Quebec, Canada; 2 DeafBlind Ontario Services, Newmarket, Ontario, Canada; 3 European Deafblind Network (EDbN), Barcelona, Catalonia, Spain; University of Birmingham, UNITED KINGDOM

## Abstract

**Background:**

Individuals with deafblindness experience a combination of hearing and vision impairments. The World Health Organization has developed a global framework referred to as the International Classification of Functioning, Disability and Health (ICF) to describe health and functioning. From the full ICF classification, a selection of categories, referred to as ICF Core Sets, provide users with a tool to describe functioning and disability in specific health conditions. There has been no ICF Core Set created for deafblindness. Given that core sets are instrumental in improving clinical practice, research, and service delivery, the aim of this study is to develop an ICF Core Set for deafblindness.

**Methods:**

As part of the preparatory phase in the ICF Core Set development, there are four studies that will be conducted. This includes the [1] systematic literature review that examines the researcher’s perspective, [2] qualitative study focusing on the individuals with deafblindness experience, [3] experts survey that looks at health professional’s perspective, and [4] empirical study that examines the clinical perspective. The studies will be conducted using the principles outlined by the ICF Research Branch for the development of ICF Core Sets. The systematic literature review protocol was submitted for registration on PROSPERO CRD42021247952.

**Discussion:**

An ICF Core Set created for deafblindness will benefit individuals living with deafblindness who are often excluded from social participation, policies, and services. An ICF Core Set for deafblindness will have a significant impact on healthcare professionals, policymakers, researchers, service providers and individuals with deafblindness by facilitating communication among all stakeholder to support the functioning of those with deafblindness.

## Introduction

Deafblindness, also known as dual sensory loss, is a combination of hearing and vision disability in the same individual [1, 2]. Representing between 0.2% to 2% of the world’s population, persons with deafblindness are a very diverse group [1]. Since deafblindness is less well-known, individuals with deafblindness struggle to obtain the right support and are often excluded from both development and disability programmes [1]. People with deafblindness frequently experience participation barriers and social isolation due to environmental factors such as lack of accessibility and availability of adaptive equipment and communication supports [1, 3]. Thus, advancing the knowledge of this complex disability is necessary to improve the functioning and quality of life of individuals with deafblindness.

The World Health Organization (WHO) developed an international classification framework to describe health and functioning, referred to as the International Classification of Functioning, Disability and Health (ICF) [4]. As of May 2001, 191 member states have officially endorsed the ICF [5]. In contrast to the International Classification of Diseases (ICD), an etiological framework for diseases, disorders, and other health conditions [4], the ICF was developed to focus on health and functioning of an individual with the disability [4, 6].

The ICF aims to capture the functionality of individuals in society despite their impairments while establishing a scientific ground to understand and develop a common language to describe health [6]. Since its inception, the framework has been used for various reasons, including statistical, research, clinical, social policy, and educational purposes [6]. For instance, at the policy level, the framework can help provide a consistent definition for the disability that can be used to develop eligibility criteria for disability pensions, accessibility and accommodation policies, or in creating regulations for assistive devices [4, 7–9].

This classification system is organized hierarchically into two main parts: functioning and disability, and contextual factors [10]. These two parts are further categorized into four components: [1] body structure and functions, [2] activities and participation, [3] environmental factors and [4] personal factors [4, 6, 10, 11]. The first three components are made of a variety of chapters, and each chapter contains several categories that can be further classified into four levels [11] whereas the fourth component has not been classified in detail.

There are over 1,400 ICF categories, making it impractical for use in everyday practice [10, 12]. Therefore, a smaller set of ICF categories, referred to as an ICF core set, can be created for specific health conditions or patient groups [10, 13, 14]. An ICF Core Set is a choice of categories from the full ICF classification that provides a practical system for describing functioning and disability in specific health conditions [15]. There are three types of ICF Core Sets. First is the Comprehensive Core Sets that includes ICF categories that reflect the entire spectrum of typical problems that individuals with a specific health condition may experience [10]. Typically, it is used as a detailed checklist to guide practitioners for a thorough and interdisciplinary assessment of functioning [10]. Secondly, the Brief Core Set, derived from the Comprehensive Core Set, is composed of the most relevant ICF categories used for brief assessments of functioning [10]. It is commonly used in clinical and epidemiological settings to describe the functioning of the health condition during brief assessments [10]. Lastly, the Generic Set is commonly used for health statistics or public health contexts and allows for comparability of functioning across different conditions [10]. On its own, the Generic Set provides an overview of the patient’s functioning [10].

The Core Sets have been developed for over thirty health conditions like neurological, respiratory and cardiovascular conditions, mental health, and other health conditions, including hearing loss [12–14, 16–19]. To date, there has been no ICF Core Set developed for deafblindness. An ICF Core Set for deafblindness can have a significant impact on healthcare professionals, policymakers, researchers, service providers and individuals with deafblindness by focusing on the description of the functioning of those with deafblindness in specific contexts. Due to the combined sensory impairment, persons with deafblindness are more likely to experience activity limitations and be excluded from social participation, face barriers in accessing services, and in receiving accessible information [1, 3, 20]. An ICF Core Set can help users understand how the impairments affect the functioning of individuals and the challenges they face within society. In turn, this can significantly improve the functioning and quality of life of those living with deafblindness at the individual, institutional, and societal levels [4].

### Aim of protocol

The purpose of this protocol is to describe the multi-step methodological approach that will be conducted to complete the preparatory phase of the ICF Core Set development for deafblindness. As part of this phase, a systematic literature review, qualitative study, experts survey, and empirical study will be performed.

## Methods

The Core Set on deafblindness will be developed following the principles outlined by the World Health Organization (WHO) and the ICF Research Branch for the development of ICF Core Sets [12]. The Core Sets are developed in three phases: the preparatory phase, the International ICF consensus conference, and the final phase is the implementation of the first version of the ICF Core Sets [12]. During the preparatory phase, the health condition is examined using four different perspectives. This includes a systematic literature review (researcher perspective), followed by an empirical multicentre study (clinical perspective), qualitative study (patient perspective), and experts’ study (health professional’s perspective) [12].

### Systematic literature review (Researcher perspective)

The systematic literature review protocol has been submitted for registration on PROSPERO CRD42021247952. The Preferred Reporting Items for Systematic Reviews and Meta-Analysis Protocols (PRISMA-P) was used to develop this protocol for the systematic literature review stage (see S1 Checklist) [21]. All available data will be shared publicly once the study is completed and published. The four stages include: identify articles that examine functioning in deafblindness, identify descriptors used to explain and evaluate functioning in deafblindness, locate and link meaningful concepts to existing ICF categories, and data analysis. Four members of the research team (AP, AJ, RM, and WW) have received training in developing a Core Set from the WHO’s ICF Research Branch in March 2021. The review study will be completed under the guidance of the Advisory Committee consisting of internationally renowned researchers and clinical experts in the field of deafblindness.

This review is guided by the research questions:

1. What are the aspects of functioning that are described or evaluated in the scientific literature on deafblindness?
2. What are the outcome measures as well as the study and population characteristics of the included studies?

Following Population, Context, and Concept (PCC) [22], this review defines the *population* as persons with deafblindness with a “distinct disability arising from a dual sensory impairment of a severity that makes it hard for the impaired senses to compensate for each other” [1]. There are no restrictions on the type or severity of deafblindness, age of onset, or the age of participants. Individuals with deafblindness can be born deafblind after diagnosis with conditions like Congenital Rubella Syndrome, CHARGE syndrome, or acquire deafblindness due to Usher Syndrome/accident/injury or acquire the disability with age [1, 23]. Studies examining either visual or hearing impairment only are not included in this review.

The *concept* of functioning is described as one of the components identified by ICF (body function, structure, participation, activities, and environmental factors). While the temporal *context* of this review is scientific literature published in the last decade, the spatial context is research that focuses on any aspects of functioning of individuals with deafblindness. The intended outcomes are aspects of functioning of individuals with deafblindness that are described or evaluated and all the outcome measures or instruments in the scientific literature on deafblindness.

Following the ICF Core Set development guideline by the ICF Research branch, we will focus on research conducted within the past decade from the review date, beginning March 2011 up to March 2021 [12]. This review will include studies in all languages. Studies with less than 5 participants will be excluded. As suggested by the guideline, prevention, psychometric, phase II clinical trials, economic evaluation studies, and genetic studies as well as studies involving animal experiments will be excluded [12]. Furthermore, study protocols, literature reviews, letters, comments, and editorials are to be excluded [12]. Table 1 presents the inclusion and exclusion criteria.

**Table 1:** Inclusion and exclusion criteria.

Inclusion Criteria	Exclusion Criteria
Studies with primary diagnosis as deafblindness or dual sensory impairment (DSI)	Studies related to non-disabled populations or other forms of disabilities such as hearing impairment or vision impairment only
Randomized control trials, clinical controlled trials, cross-sectional studies, observational studies, qualitative studies	Studies that focus on genetics, psychometric, preventative, phase II clinical trials, laboratory experiments involving animals
Studies published from March 2011 to March 2021	Literature reviews, study protocols, letters, editorials, and comments
Studies from all countries and languages	

#### Data collection

The research team developed a search strategy in consultation with a trained librarian (see S1 Appendix). Scholarly electronic databases to be searched include MEDLINE, EMBASE, CINAHL, Global Health, PsycINFO, ERIC, Web of Science using the Preferred Reporting Items for Systematic Review and Meta-Analyses (PRISMA) [24, 25]. In addition to the scientific databases, the research team will search the Örebro University database on deafblindness to ensure the comprehensiveness of the search. The first 100 hits in the Google Scholar on deafblindness and functioning will also be screened. Moreover, the journals ‘*Journal of Visual Impairment and Blindness’*, *‘Journal of Deafblind Studies on Communication’* and the ‘*British Journal of Visual Impairment’* will be hand-searched, given that not all articles in these relevant journals are indexed. The reference manager, Mendeley Desktop Version 1.19. 6 (Mendeley, London, United Kingdom), will be used to transfer search results. The screening tool, Covidence (Veritas Health Innovation, Melbourne, Australia), will be used to conduct abstract and full text level screening.

A team of 50 volunteers (recruited through the network support of the Deafblind International) from around the world will be trained to assist in various steps of the review. The volunteer team will be provided with a series of one-hour training session on the title/abstract screening and full-text screening using Covidence, data extraction and ICF linking by the research team. To ensure inter-reviewer reliability, the volunteer reviewers will practice screening of at least 20 sources to familiarize themselves with each screening step—title/abstract and full-text level. Then, two independent reviewers will screen each article at the two levels following the requirements of the inclusion criteria (See Table 1). If a substantial number of articles remain following abstract screening, a random sampling process of the full text review will occur. A senior reviewer (WW) will be involved in resolving conflicts. Studies not examining one of the identified outcome measures mentioned above will be excluded. Each study will be tagged with a label indicating the age group of the population—children (ages 0–17), adults (ages 18–64), and older adults (ages 65+). A PRISMA flow chart will be utilized to outline the stages in study selection.

#### Data analysis

The research team will develop a charting template that includes descriptors like publication details, type of study, location, age, sex, diagnosis, aetiology, country, information about the population, instruments, and types and frequency of outcome measures. Two independent trained reviewers of the volunteer team will extract each included study. Two members of the research team (AP and AJ) will independently review the extracted data from the studies. Once all data have been extracted, the team will meet to review the included studies and discuss differences in, and harmonize perspectives of, study inclusion. Findings will be depicted in tables and figures and interpreted using qualitative thematic analyses [26]. The research team will discuss collectively how to best identify the presentation of the identified themes.

Following data extraction, the full text of each included article and its outcome measures will be analysed to identify the meaningful concept. Meaningful concepts are ideas or information that are contained within a statement [27]. The meaningful concept will be linked to the ICF categories using the established ICF linking rules by two reviewers independently. There are three specific linking rules developed for health status measures, one linking rule on technical/clinical measures and interventions, and eight linking rules to be used for all outcome measures/interventions [13, 28–30].

In order to link, each meaningful concept needs to be interpreted to determine its meaning in the context of the situation. The researcher will identify the correct components and chapters the concept belongs to. Based on the description and inclusion/exclusion of each category’s level, the researcher will link to the correct ICF category. Two researchers will link the same meaningful concept. Any discrepancies will be discussed as a group and alterations to the linking method will be performed if needed. Once all linking has been completed, a consensus meeting will be held to identify any further discrepancies. As suggested by the ICF research branch guide, categories that are found in at least 5% of the publications will be represented in the list of candidate categories [12]. Lastly, a frequency analysis will be conducted on the linked categories [12]. ICF categories that appear in a publication multiple times will only be counted once [12].

### Qualitative study (Deafblind population)

Ethics approvals for the experimental studies listed below will be obtained through the institutional review boards at the affiliations of the respective co-investigators. The goal of the qualitative study is to capture the unique experiences of individuals living with deafblindness as they relate to functioning [12]. The research team will conduct focus groups with persons with deafblindness using seven open-ended questions, as suggested by the ICF [12]. There will be at least one focus group per each of the six WHO regions. The research team prefers to have a minimum of one focus group per country because of the diverging contextual factors affecting those with deafblindness. The global network of Deafblind International and the European Deafblind Union will be employed for study recruitment and data collection.

#### Study population

Participants must be aged 18 or older, able to provide informed consent, and be willing to share their experiences related to functioning and disability. Purposive sampling will be used to recruit participants [31]. Participants can be individuals with deafblindness and/or the parents/carers of an individual with deafblindness. We aim to include participants with different types of deafblindness (congenital, acquired, or age-related) in the focus groups. Study participants will be divided into three age groups based on the assumption that persons of similar ages and life phases would stimulate group interaction and discussion.

#### Data collection

A series of focus groups will be organized according to age groups (group 1: 18–40; group 2: 41–60; and group 3: 61 years and above) until qualitative data saturation is achieved [32]. Focus groups are used to generate rich data from the collective views and in-depth discussion among participants who may have similar or divergent views or experiences [33]. In case participants with deafblindness cannot participate in focus groups such as due to communication barriers, time availability, or personal preference, one-on-one semi structured interviews will be organized to facilitate participation and to capture and represent their experiences [20, 32]. An intervenor/interpreter will be present to help facilitate participation and will receive an orientation session to provide an overview of the research terminologies that will be used. Diverse accessible communication modes (i.e., use of multimedia, large prints, face to face discussion, accessible audio formats) will be used to enable participation in the research and to make questions accessible to participants [34]. Each focus group will have a moderator and at least one assistant. The moderators will be responsible for facilitating the question process and will be familiar with common problems experienced by people with deafblindness. The assistant will be responsible for taking field notes and monitoring recorders. Both the moderator and group assistant will have received ICF training prior to conducting the focus group.

Participants will complete a Case Record Form that will be developed containing questions about socio-demographic information and their deafblindness. Once all participants provide informed written consent, the focus group sessions will be audio recorded. The moderator will provide a brief introduction and ask the seven open-ended questions. Each question will relate to the one of the ICF components—body functions, body structures, activities and participations, environmental factors, and personal factors [12]. Table 2 presents the questions that will be asked in the qualitative study. After each ICF component discussion, a summary of the main results will be provided to the focus group participants to review, verify, and amend the findings, as part of enhancing rigor of the data [35].

**Table 2:** Qualitative study questions.

ICF Components	Open-Ended Questions
**Body Functions**	How does your deafblindness affect the way your body and mind work?
**Body Structures**	Which parts of your body does your deafblindness give you problems?
**Activities and Participation**	How does your deafblindness affect the things you can and cannot do in your everyday life?
**Environmental factors facilitator**	What and/or who in the environment where you live and work/go to school is helpful and supportive in your everyday life with deafblindness?
**Environmental factors barriers**	What and/or who in the environment where you live and work/go to school make everyday life with deafblindness difficult for you?
**Personal factors**	When you think about yourself and the person you are, what helps you to handle your everyday life with deafblindness?

#### Data analysis

Interviews and focus groups will be audio-recorded, transcribed, and managed using NVivo software (QSR International, Massachusetts, USA). The qualitative data will be analysed using directed content analysis [36]. Two reviewers will review the same transcript. The statements that relate to functioning will be divided into meaningful concepts. The reviewers will compare the meaningful concepts identified and disagreement will be resolved by a third senior reviewer. Following this process, the reviewers will link each of the identified meaningful concept independently. Upon completion of the linking process, the reviewers will compare the ICF categories to identify any discrepancies. Any disagreements will be resolved by a third reviewer to develop a final list of categories. Following analysis of each focus group session transcript, the research team will determine whether further participants need to be recruited to perform other focus group sessions. This is to ensure saturation of data results, in which linking of meaningful concepts in two consecutive focus groups results in no additional 2^nd^ level ICF categories [12].

### Expert survey (Health professional perspective)

The expert survey aims to identify the perspective of experts working with individuals with deafblindness from all six WHO regions [12].

#### Study population

Experts participating in the survey must have a minimum of two years of experience working with individuals with deafblindness. Specifically, this study is interested in including experts that work as occupational therapists, intervenors, rehabilitation specialists, medical doctors, nurses, social workers, optometrists, and audiologists. The expert survey will be available in English only. Therefore, experts must be able to understand and respond in English to participate. The team will recruit experts from international organizations, not-for profit organizations, academic centres, hospitals, DeafBlind societies/chapters, social media, and through personal networks. Each expert will be asked to include their discipline and WHO region to ensure that a global perspective and from a variety of fields is captured in the survey data. Like the qualitative study, due to the heterogenous nature of deafblindness, the team aims to have at least one expert per country. However, due to the lack of awareness of deafblindness in some countries, this study aims to have an overall target sample of 100 experts. Each prospective expert’s data will be entered into an Excel file to develop an expert pool. Additionally, to prevent oversaturation of one WHO region over another, the team will aim to have a similar number of experts per region. Prospective experts will be sent an email with an invitation to participate in the survey and will have 2–3 weeks to accept the invite.

*Data collection*. An internet-based survey of open-ended questions will be designed to understand the problems and barriers encountered by individuals with deafblindness from the perspective of experts in the field [12]. Surveys will be emailed to experts who accepted the invitation only. The first section will include a section for the expert to provide details about their profession, experience working with individuals with deafblindness, number of years working in the field, and sociodemographic information [12, 37]. The second section will ask six open-ended questions related to functioning of individuals with deafblindness. Table 3 presents the questions that will be asked in the expert study regarding functioning [12, 37]. Experts will be asked to write detailed responses. The last section will be a privacy and consent form [37].

**Table 3:** Expert study questions.

ICF Components	Open-Ended Questions
**Body Functions**	From your experience working with individuals with deafblindness, how does deafblindness affect the way their body and mind works?
**Body Structures**	From your experience working with individuals with deafblindness, which parts of their body does deafblindness gives them problems?
**Activities and Participation**	From your experience working with individuals with deafblindness, how does deafblindness affect the things they can and cannot do in your everyday life?
**Environmental factors facilitator**	From your experience working with individuals with deafblindness, what and/or who in the environment is helpful and supportive in their everyday life with deafblindness?
**Environmental factors barriers**	From your experience working with individuals with deafblindness, what and/or who in the environment is difficult in their everyday life with deafblindness?
**Personal factors**	From your experience working with individuals with deafblindness, what personal characteristics support their daily functioning with deafblindness?

#### Data analysis

Survey responses will be analysed by conducting a thematic or content analysis [26, 36]. Like the previous two studies, each text passage will be divided into meaningful concepts and then linked to ICF categories. Repeated ICF categories will only be counted once for each expert. Lastly, categories found in at least 5% of the publications will be represented in the list of candidate categories for the international consensus conference.

### Empirical cross-sectional study (Clinical perspective)

Lastly, a cross-sectional study will be conducted to identify the common issues experienced by individuals with deafblindness that are documented in a clinical setting, such as a primary care practice or a community health centre [12].

#### Study participants

Study participants from the qualitative study will be given the opportunity to participate in the empirical study and further participants will be recruited through organizations and primary care practices. Participants must be aged 18 or older and diagnosed with deafblindness. The team will aim to recruit persons with deafblindness via different aetiologies. Additionally, clinicians from the expert survey will be given the opportunity to join the empirical study. If there are not enough clinicians from the expert survey, clinicians will be recruited from international organizations, associations, hospitals, social media, and through personal networks.

#### Data collection

Clinicians will conduct semi-structured interview with patients with deafblindness. The clinician will use the extended ICF checklist V2.1a to conduct the interview. This checklist includes information about socio-demographic characteristics and 125 ICF categories that WHO identified as important in a clinical practice [38, 39]. Additionally, the systematic literature review’s top three commonly used instrument’s ICF categories will be included in the checklist [12]. Clinicians will rate each of the ICF categories included in the checklist and instruments based on the extent at which the environmental factor is a facilitator or a barrier for the client with deafblindness [12]. The qualifiers range from 0–4; 0 meaning no impairment and 4 meaning complete impairment [38]. Additionally, qualifiers 8 and 9 are available to code for “not specified” or “not applicable” respectively [38].

#### Data analysis

Descriptive statistics will be used to analyse the qualifiers of the ICF categories from the checklist and instrument [12]. From this analysis, a list of ICF categories will be developed. An ICF category must appeared in at least 20% of the total patients included in the study to include it in the list of candidate categories [12].

From the four studies conducted, four lists of candidate categories will be identified. These candidate categories will be combined to create a preliminary comprehensive ICF Core Set for deafblindness [39].

### International consensus conference

In line with the requirements of the World Health Organization ICF Research branch [12], an international consensus conference will complete the process for the development of the core set for deafblindness. This consensus conference is currently planned to be held as a pre-conference event, the one-two day before the 18th Deafblind International World Conference, in Ottawa, Ontario, Canada in July 2023. A maximum of 24 team members and experts, representing equal distribution across professional disciplines and WHO world regions, will discuss a synthesis of the 4 previous phases. They will discuss the candidate categories that have been identified during the preparatory studies and will engage in an iterative decision-making process to finalize the final core set, based on affirmation percentages. ICF categories that were affirmed by at least 75% of the participants will automatically be included in the Comprehensive ICF-CS.

### Implementation of ICF—Core Sets

Lastly, the final phase is the implementation of the first version of the ICF Core Set for deafblindness into practice. This stage will help determine whether the ICF categories included in the Core Set for deafblindness captures all problems experienced by individuals with deafblindness globally and identify important categories that are missing from the Core Set [12, 40, 41].

## Discussion

With the growing population affected by deafblindness, there is a greater demand for policies and services that meet their unique needs. The creation of the Core Set for deafblindness will be significant in improving the lives of those living with deafblindness within different sectors. The Core Set will establish a common language and provide users with a stronger understanding of the functional effect of the disability has on individuals. Furthermore, given the lack of understanding and knowledge of deafblindness as a unique disability means that an ICF Core Set can raise awareness of the condition and contribute to public education. This can help clinicians, administrator and other stakeholders with providing tailored care to their patients or inform policies that can ensure accessibility to health services for the deafblind population. As the first phase of the development of the ICF Core Sets for deafblindness, these studies will be significant in identifying the functioning of individuals with deafblindness and the ICF categories to be used in the Core Set. Moreover, the global collaboration amongst researchers, experts, clinicians, and individuals with deafblindness involved in this project will assist in building greater awareness and a global network in the field of deafblindness. The various studies as part of the core sets development will provide an excellent opportunity to generate scientific evidence in the field of deafblindness and mainstream this less well-known, but very significant health condition in health services and policies research agendas across the globe. Once the studies are completed, results will be published in scientific journals and presented in various national/international conferences.

### Limitations

Given that the primary purpose of this systematic literature review is to identify functional outcomes and the study follows rigorous scientific protocol proposed by the WHO and the ICF Research Branch, the literature will not be critically appraised to identify methodological rigor or study quality. Furthermore, only articles from the last decade will be included in the systematic literature review. Therefore, possible functional concepts that were explored at an earlier date will not be captured. However, the selected ICF categories must appear in 5% of recent publications, meaning the exclusion of these articles may not have a significant impact. There may be limitations in recruitment of individuals with deafblindness in certain countries, meaning that the perspective of individuals can be missed. For example, a person with deafblindness’ functioning may significantly vary if s/he lives in a country with limited resources compared to an individual with access to an abundance of resources. This can be significant in the qualitative and empirical study where individuals with deafblindness will be recruited.

## Supporting information

S1 Appendix Search strategy. (DOCX)

S1 Checklist PRISMA-P 2015 checklist. (DOCX)
