# Supplementary material for: The development of the International Classification of Functioning, Disability and Health Core Sets for deafblindness: A study protocol
Source: PLoS One. 2021 Dec 14;16(12):e0261413. doi: 10.1371/journal.pone.0261413 (PMC8670675; doi:10.1371/journal.pone.0261413)
Supplement: S1 Appendix — (DOCX) [file pone.0261413.s001.docx]

**Appendix 1: Search Strategy**

EMBASE - 1974 to 2021 March 2021:

1. deafblindness/
2. ((sensory or sensation) adj (impair* or loss* or disorder? or deficienc* or dysfunction? or defect?) adj10 (dual or double or multi or multiple)).tw.
3. (sensory dysfunction/ or (exp hearing disorder/ and exp visual disorder/)) and (dual or double or multi or multiple).tw.
4. (((vision or visual*) adj (impair* or loss* or disorder? or deficienc* or dysfunction? or defect?) adj10 (dual or double or multi or multiple)) and ((hearing or auditory) adj (impair* or loss* or disorder? or deficienc* or dysfunction? or defect?) adj10 (dual or double or multi or multiple))).tw.
5. (Deaf* and blindness* and (dual or double or multi or multiple)).tw.
6. (dual impairment? or dual dysfunction? or "hearing and vision loss*" or "Vision and Hearing Loss*" or deafblind* or deaf-blind* or Deafness Blindness or Blind-Deaf* or Deaf-Mutism-Blind or "hearing and visual impairment?" or "hearing and visually impaired" or "hearing and visual disabilit" or "hearing and visual disabilit" or "hearing and visually disabled" or "vision and hearing impairment?" or "visually and hearing disabled" or "vision and hearing disability" or "vision and hearing disabilities" or "vision and hearing dysfunction" or "vision and hearing defect").tw.
7. (((Usher or Hallgren or Wolfram or pharc or didmoad or alstrom or charge or refsum or norrie or mohr tranebjaerg or cockayne or stickler or melas or branchio oculo facial or alport) adj Syndrome?) or Dystrophia Retinae Pigmentosa-Dysostosis Syndrome? or Retinitis Pigmentosa Deafness Syndrome? or Deafness-Retinitis Pigmentosa Syndrome? or (Retinitis Pigmentosa and Congenital Deafness*)).tw.
8. 1 or 2 or 3 or 4 or 5 or 6 or 7
9. limit 8 to yr="2011 -Current"
